# *Plasmodium falciparum* Hep1 Is Required to Prevent the Self Aggregation of PfHsp70-3

**DOI:** 10.1371/journal.pone.0156446

**Published:** 2016-06-02

**Authors:** David O. Nyakundi, Loyiso A. M. Vuko, Stephen J. Bentley, Heinrich Hoppe, Gregory L. Blatch, Aileen Boshoff

**Affiliations:** 1 Biotechnology Innovation Centre, Rhodes University, Grahamstown 6140, South Africa; 2 Department of Biochemistry and Microbiology, Rhodes University, Grahamstown 6140, South Africa; 3 Biomedical Biotechnology Research Unit, Department of Biochemistry and Microbiology, Rhodes University, Grahamstown, South Africa; 4 College of Health and Biomedicine, Victoria University, Melbourne, Victoria 8001, Australia; University of Pittsburgh, UNITED STATES

## Abstract

The majority of mitochondrial proteins are encoded in the nucleus and need to be imported from the cytosol into the mitochondria, and molecular chaperones play a key role in the efficient translocation and proper folding of these proteins in the matrix. One such molecular chaperone is the eukaryotic mitochondrial heat shock protein 70 (Hsp70); however, it is prone to self-aggregation and requires the presence of an essential zinc-finger protein, Hsp70-escort protein 1 (Hep1), to maintain its structure and function. PfHsp70-3, the only Hsp70 predicted to localize in the mitochondria of *P*. *falciparum*, may also rely on a Hep1 orthologue to prevent self-aggregation. In this study, we identified a putative Hep1 orthologue in *P*. *falciparum* and co-expression of PfHsp70-3 and PfHep1 enhanced the solubility of PfHsp70-3. PfHep1 suppressed the thermally induced aggregation of PfHsp70-3 but not the aggregation of malate dehydrogenase or citrate synthase, thus showing specificity for PfHsp70-3. Zinc ions were indeed essential for maintaining the function of PfHep1, as EDTA chelation abrogated its abilities to suppress the aggregation of PfHsp70-3. Soluble and functional PfHsp70-3, acquired by co-expression with PfHep-1, will facilitate the biochemical characterisation of this particular Hsp70 protein and its evaluation as a drug target for the treatment of malaria.

## Introduction

Heat shock protein 70 (Hsp70) family members are present in all organisms and are the most highly conserved heat shock protein family [1]. Hsp70 proteins function as molecular chaperones, and are involved in various cellular processes such as protein folding and assembly of nascent polypeptides, refolding aggregated proteins, protein translocation across membranes, protein degradation and controlling the activity of regulatory proteins [2–5]. Allosteric coupling between the N-terminal ATPase domain and the C-terminal substrate binding domain is essential for the function of Hsp70 and is mediated via the inter-domain linker [6–7]. ATP binding and hydrolysis leads to conformational changes in the two domains and this regulates substrate affinity [8]. The functions of Hsp70 are regulated by co-chaperones, such as J-proteins (also called Hsp40 or DnaJ), and by GrpE-like nucleotide exchange factors [9]. Due to their crucial role in proteostasis, Hsp70 members are present in almost all cellular compartments [10].

The function of the mitochondria is critically dependent on mitochondrial Hsp70 (mtHsp70) [11] and it plays a major role in the translocation of nuclear encoded proteins across the mitochondrial membranes and folding of proteins in the matrix [12–13]. Ssc1, Ssq1 and Ssc3/Ecm10 are the Hsp70 proteins found in the mitochondria of yeast, with Ssc1 being the major Hsp70 [14–15]. Protein folding by Ssc1 in the matrix is regulated by Mdj1 (the only Type I J-protein in the mitochondria) which delivers substrate and stimulates the ATPase activity [16–17]. Unexpectedly, mtHsp70 has the propensity to self-aggregate and it requires an additional essential regulator, Hep1 (Hsp70 escort protein), to maintain its functional state [18]. Hep1 orthologues are conserved in many eukaryote species including protozoa, but not in prokaryotes [19]. Only Ssc1 and Ssq1 produce aggregation-prone conformers of the ATPase domain that bind to Hep1 [20]. Yeast cells deprived of Hep1/Zim17/Tim15 accumulate insoluble mtHsp70 [18] and generally exhibit mitochondrial defects similar to those observed upon mtHsp70 deletion [18–20].

Hep1 is a zinc-finger protein with one tetracysteine motif that is part of the zinc finger domain [21]. The binding of zinc ions to Hep1 in the mitochondria is critical for its function [19]. Hep1 only binds to nucleotide-free mtHsp70 and is released upon nucleotide binding [18, 20, 22]. The N-terminal ATPase domain of mtHsp70 in association with the interdomain linker is prone to aggregation, while the ATPase domain and C-terminal substrate binding domain are both soluble when expressed separately [18, 20]. The inter-domain linker attached to the ATPase domain is the minimal binding entity required by Hep1 to keep mtHsp70 soluble and active [20, 21, 23].

The *P*. *falciparum* genome encodes six Hsp70 orthologues, and only PfHsp70-3 has been predicted to localise in the mitochondria [24]. Plasmodial Hsp70 proteins and their interactions with co-chaperones have received attention as potential avenues for drug targeting as they play an integral part in the survival and pathology of the parasite. Selective modulation by small molecules of *P*. *falciparum* Hsp70 proteins has been demonstrated [25], as well the modulation of the Hsp70/J-protein partnership [25–26]. Little is known about the role of PfHsp70-3 in the parasite, and a protein translocation model was previously described by van Dooren et al. [27]. More recently, an overview of the proposed roles of PfHsp70-3 and its co-chaperones in the mitochondria was presented by Njunge et al. [24]. The putative co-chaperones of PfHsp70-3 which form part of the import machinery are Tim44 (PF11_0265), GrpE (PF11_0258) and PfPam18 (PF07_0103) [24]. Other proposed co-chaperones of PfHsp70-3 involved in protein refolding in the matrix are Pfj1 and PFF1415c but these have not been experimentally validated [24]. PfHsp70-3 is an orthologue of Ssc1 and is predicted to localize in the parasite’s mitochondrion and play a central role in the translocation of proteins into the mitochondria and their subsequent folding in the matrix [24]. Our results show that PfHsp70-3 is indeed insoluble when heterologously produced in *E*. *coli* cells. We identified a putative Hep1 orthologue in *P*. *falciparum*, and provide experimental evidence that PfHep1 prevents the self-aggregation of PfHsp70-3 that is required for structural and functional activities. We also examined the abilities of PfHsp70-3 and PfHep1 to function as holdases and suppress protein aggregation, and demonstrate that the zinc ion in the zinc finger domain is essential for stabilising the structure of PfHep1.

## Materials and Methods

### Primary structure sequence analysis and homology modelling of the zinc-binding domain (ZBD) of PfHep1

The protein domain mapping for PfHep1 was conducted using the online programs SMART 7 (Simple Modular Architecture Research Tool; http://smart.embl-heidelberg.de/; [28]), and Prosite (http://prosite.expasy.org/; [29]). In order to predict the subcellular localisation of PfHep1 and PfHsp70-3 a number of online programs that included NucPred (http://www.sbc.su.se/~maccallr/nucpred/cgi-bin/single.cgi; [30]), MitoPROT (http://ihg.gsf.de/ihg/mitoprot.html; [31]), MultiLoc (http://abi.inf.uni-tuebingen.de/Services/MultiLoc; [32], SignalP version 4.1 (http://www.cbs.dtu.dk/services/SignalP/; [33]), and WoLF PSORT (http://www.genscript.com/wolf-psort.html.; [34]) were used. The primary amino acid sequence of PfHep1 and other selected well-characterised Hep1 orthologues were obtained from PlasmoDB v4.4 (http://plasmodb.org/plasmo/; [35]), and the NCBI database. Alignment was conducted using MAFFT (http://www.ebi.ac.uk/Tools/msa/mafft/; [36]). The zinc-binding domain structure of PfHep1 was modelled using the online Swiss Model server [37]. The solution structure of Tim15c (yHep1) solved by NMR (PBD accession number 2EZZ) was used as the template [21]. The model was rendered using PyMol [38].

### Construction of *E*. *coli* expression plasmids encoding PfHsp70-3 and PfHep1

*E*. *coli* codon-optimized versions of the Hsp70-3 coding sequence (PlasmoDB accession number: PF3D7_1134000) (41 – 622aa) and PfHep1 coding sequence (PlasmoDB accession number: PF3D7_1420300) (15–302 aa), both lacking the mitochondrial signal peptides, were synthesized by the GenScript Corporation (Piscataway, New Jersey, USA) and inserted into a pQE30 expression vector (Qiagen, Germany) to create the pQE30-PfHsp70-3 and pQE30-PfHep1, while the pACYCDuet1 expression vector was used to create pACYCDuet1-PfHep1.

### Expression and purification of PfHep1

*E*. *coli* M15 (pREP4) cells were transformed with pQE30-PfHep1 and grown at 37°C in 2x YT medium supplemented with 100 μg/ml ampicillin and 50μg/ml kanamycin and grown to mid-logarithmic phase (A_600_ 0.4–0.6). Protein production was induced by addition of 0.1 mM IPTG. Cells were harvested prior to induction and at hourly intervals post induction for 5 hours and overnight. Cells were harvested by centrifugation (13000×g; 2 min) and re-suspended in PBS buffer. Protein production levels were evaluated using SDS-PAGE and Western blot analysis. For recombinant protein purification, cells were harvested at the fourth hour post induction and the harvested cells were re-suspended in lysis buffer (10 mM Tris-HCl, pH 7.5, 300 mM NaCl, 10 mM imidazole, 1 mM PMSF, 1 mg/ml lysozyme) and frozen at -80°C overnight. The cells were then thawed on ice and sonicated at 4°C in the presence of 3% sarcosyl (Sigma-Aldrich, Germany). The lysed cells were centrifuged (16000×g, 40 min, 4°C) and the soluble supernatant fractions were mixed with cOmplete His-tag purification resin (Roche, Germany) and allowed to bind overnight at 4°C with gentle agitation. The resin was then pelleted by centrifugation (5000×*g*; 2 min) to remove unbound proteins and washed three times using wash buffer (100 mM Tris-HCl, pH 7.5, 300 mM NaCl, 50 mM imidazole) to remove non-specific contaminants. The bound protein was eluted by re-suspending the resin in elution buffer (10 mM Tris-HCl, pH 7.5, 300 mM NaCl, 750 mM imidazole). The eluted protein was extensively dialysed using SnakeSkin dialysis tubing (Pierce-MWCO 10,000; Thermo Scientific, USA) into dialysis buffer (10 mM Tris, pH 7.5, 100 mM NaCl, 0.5 mM DTT, 10% (v/v) glycerol, 50 mM KCl, 2 mM MgCl_2_), and concentrated against PEG 20000 (Merck, Germany). The efficacy of the purification process was assessed using SDS-PAGE and western analysis using mouse monoclonal anti-His primary antibody and HRP-conjugated goat anti-mouse IgG secondary antibody (Santa Cruz Biotechnology, USA). Chemiluminescence-based protein detection was achieved using the Clarity^TM^Western ECL blotting kit (Bio-Rad, USA) as per the manufacturer’s instructions, and captured with a Chemidoc chemiluminescence imaging system (Bio-Rad, USA). The protein concentration for the purified proteins was quantified using the Bradford’s assay (Sigma-Aldrich, USA) with BSA as the standard. A fraction of the purified PfHep1 protein was dialysed extensively against buffer (50 mM Tris-HCl pH 7.5, 300 mM NaCl, 1 mM DTT, and 1 mM PMSF) containing 200 mM EDTA. The protein was then further dialysed in buffer without EDTA.

### Co-expression and co-production of PfHsp70-3 and PfHep1 in *E*. *coli*

Co-production of PfHep1 and PfHsp70-3 in *E*. *coli* was conducted in order to functionally assess the escort activity of PfHep1. *E*. *coli* BL21 (DE3) cells were transformed with pQE30-PfHsp70-3 in the presence of pACYCDuet1 (empty vector) or pACYCDuet1-PfHep1 and grown at 37°C in 2x YT broth supplemented with 100 μg/ml ampicillin and 34 μg/ml chloramphenicol to mid-logarithmic phase, and protein production was induced by adding 0.1 mM IPTG. Cells were harvested prior to induction and at hourly intervals post induction for 5 hours and overnight. The harvested cells were centrifuged (13000×g; 2 min) and re-suspended in PBS buffer. Protein production levels of PfHsp70-3 in the presence or absence of PfHep1 were evaluated using SDS-PAGE and western analysis. The procedure for purification of the co-expressed PfHep1 and PfHsp70-3 was carried out as described for PfHep1 except that cells were harvested 5 hours post induction. For subsequent *in vitro* analyses, gel filtration was employed in order to separate the co-produced proteins. The dialysed protein solution was filtered through 0.2 μm filters and loaded into a HiPrep™ 16/60 Sephacryl™ S-200 HR column driven by an ÄKTA fast-protein liquid chromatography system (GE Healthcare, Biosciences, UK). The proteins were eluted at a flow rate of 0.5 ml/ min and 1 ml elution fractions were collected and analysed using SDS-PAGE and western analysis. The eluted proteins were concentrated against polyethylene glycol (PEG) 20000 (Merck, Germany).

### Suppression of thermally-induced PfHsp70-3 aggregation by PfHep1

An evaluation of the ability of PfHep1 to suppress the thermally-induced aggregation of PfHsp70-3 was adapted and modified from Dores-Silva et al. [39]. The suppression of PfHsp70-3 aggregation by PfHep1 was monitored by light scattering at 360 nm for 30 min at 50°C in assay buffer (50 mM Tris-HCl, 100 mM NaCl; pH 7.4). For this assay, 0.8 μM PfHsp70-3 was used with stoichiometric concentrations of PfHep1 (dialysed in the presence and absence of EDTA). An evaluation of PfHep1 and EDTA-treated PfHep1 (E-PfHep1) to self-aggregate under the assay conditions was also conducted. Each assay was conducted in triplicate and three independent experiments on independent batches of proteins were conducted. Absorbance was plotted as percentage of PfHsp70-3 aggregation subsequent to normalizing against assays with PfHsp70-3 alone.

### Suppression of thermally-induced MDH aggregation by PfHsp70-3 and PfHep1

An evaluation of the ability of PfHep1 and PfHsp70-3 to suppress the thermally-induced aggregation of MDH was adapted from Burger et al. [40]. Varying concentrations of PfHsp70-3 (0.25–1 μM), PfHep1 (0.25–1 μM) and combinations of these proteins in assay buffer (50 mM Tris-HCl, 100 mM NaCl; pH 7.5) were used to assess the abilities of these proteins to suppress the aggregation of 0.72 μM MDH by monitoring light scattering at 360 nm for 30 min at 45°C. PfHep1 alone did not self-aggregate under the assay conditions (data not shown). Absorbance was plotted as percent MDH aggregation over 30 min subsequent to normalizing against assays with MDH alone. Each assay was conducted in triplicate and three independent experiments on independent batches of proteins were conducted.

### Suppression of thermally induced citrate synthase aggregation by PfHsp70-3 and PfHep1

The ability of PfHep1 and PfHsp70-3 to suppress thermally induced aggregation of citrate synthase was adapted and modified from Lee et al. [41]. Different concentrations of PfHsp70-3 (0.25 and 1 μM), PfHep1 (0.25–1 μM) and a combination of these proteins in an assay buffer (100 mM HEPES-KOH, pH 7.5) together with 0.15 μM citrate synthase from porcine heart (Sigma-Aldrich) were used. Suppression of aggregation was determined by monitoring light scattering at 320 nm for 30 min at 45°C Absorbance was plotted as percent over citrate synthase aggregation subsequent to normalizing against assays with citrate synthase alone. Each assay was conducted in triplicate and three independent experiments on independent batches of proteins were conducted.

## Results and Discussion

### The functional domain of PfHep1 is conserved

**Fig 1:**
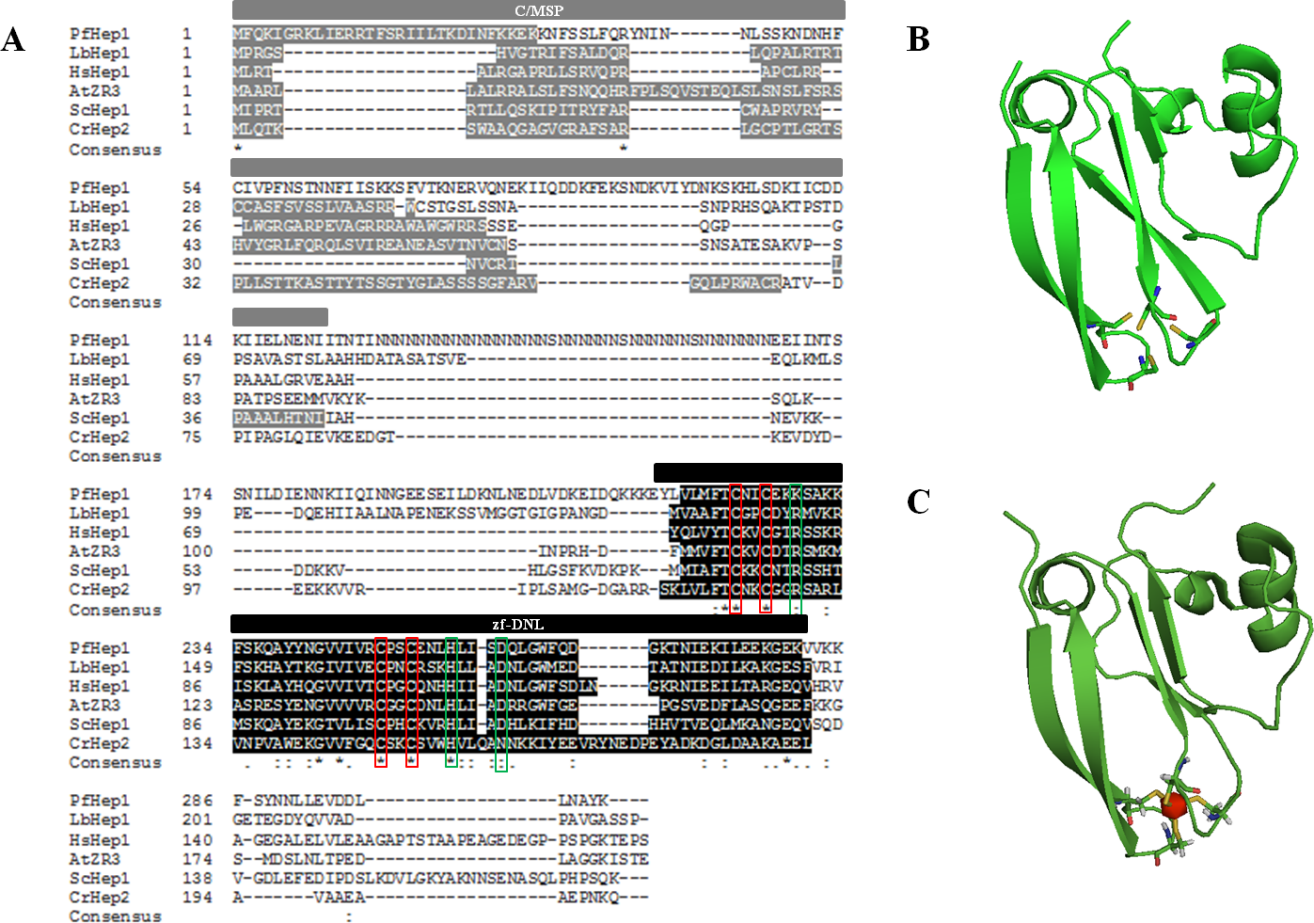
Primary structure analysis and homology model of the zinc binding domain (zf-DNL) of PfHep1. (**A**) Alignment of full-length PfHep1 with selected Hep orthologues from *Leishmania braziliensis* (LbHep1;XP_001565573.1), *Homo sapiens* (HsHep1; NM_001080849), *Arabidopsis thaliana* (AtZR3; AAO64784.1), *Chlamydomonas reinhardtii* (CrHep2; XP_001700157.1) and *Saccharomyces cerevisiae* (yHep1; NP_014089.2), where the mitochondrial/chloroplast signalling peptide (M/CSP) for each protein is shown in dark grey, and the zinc binding domain (zf-DNL) is shown in black. Degree of amino acid conservation is symbolized by the following: (*) all fully conserved residues; (:) one of the residues is fully conserved and (.) residues are weakly conserved. The conserved cysteine residues are highlighted with red boxes, and the residues implicated in facilitating interaction with their Hsp70 chaperone partner are highlighted with green boxes. (**B**) Structure of the zf-DNL of PfHep1 was modelled using the yeast Hep1 (Zim17/Tim15) structure (PBD accession no. 2E2Z) as the template and generated using the online Swiss Model program [37]. (**C**) Structure of the zf-DNL of yeast Hep1. The zinc ion is shown in red. Models were rendered using PyMol [38]. The tetracysteine motifs implicated in zinc chelation are shown as ball and stick.

Based on the observation that mitochondrial members of the plant zinc ribbon (ZR) protein family show sequence similarities to Hep1 from yeast and humans, ZR and Hep proteins were classified as part of a family consisting of five subgroups: ZR1, ZR2, ZR3, Hep1 and Hep2 [42]. The classification was based on sequence identity and sub-cellular localization of individual proteins within the cell [42]. The ZR subfamily refers to plant zinc finger proteins, ZR1 and ZR2 being plastidic and ZR3 residing within the mitochondria. The Hep subfamily refers to non-plant zinc finger proteins with Hep1 being mitochondrial and Hep2 being plastidic [42]. The putative mitochondrial targeting sequence and the zinc finger domain (zf-DNL) of the hypothetical zinc finger protein from *P*. *falciparum* (PF3D7_1420300) was identified, and based on its sequence identity and predicted subcellular localization, it was denoted as PfHep1 (Fig 1A).

Hep and ZR proteins have been observed to play roles in suppressing the self-aggregation of the respective mtHsp70 orthologues in humans [23], yeast [19, 21], Leishmania [39], green algae [43] and in *Arabidopsis thaliana* [42]. The primary structure of full-length PfHep1 was aligned with its orthologues and the putative mitochondrial or chloroplast signal peptides are highlighted for each sequence (Fig 1A). The PfHep1 sequence is asparagine-rich, with a continuous stretch from residues 128–166. In fact approximately a quarter of all amino residues found in PfHep1 are asparagines. The asparagines repeats are characteristic of the *P*. *falciparum* proteome and are often absent from heat shock proteins [44]. PfHep1 is also larger than its orthologues with 302 amino acid residues. Whilst the region upstream of the zf-DNL is longer in PfHep1 than its orthologues, the C-terminal region is slightly longer in both the hsHep1 and ScHep1 sequences (Fig 1A). There is lack of sequence conservation outside of the zf-DNL domain (Fig 1A). No functional domains, with the exception of the zf-DNL, were identified in the primary sequence of PfHep1. However, the generation of truncation mutants in LbHep1 indicated that the region upstream of the zf-DNL contributes to enhancing the solubility of LbmtHsp70 [39]. The highest sequence identity was 38% between PfHep1 and CrHep2. This is not surprising as the apicoplast, non-photosynthetic plastid, in *P*. *falciparum* is of algal origin [45]. There was approximately 25% sequence identity between PfHep1 and the remaining orthologues. Despite the low overall sequence identities between Hep proteins, the zinc-finger motifs (CXXC) that are part of the zf-DNL were found to be conserved in all of the sequences including PfHep1 and are separated by 21 amino acids (Fig 1A). Yeast mutants harbouring either a C75S or C100S mutation in the tetracysteine motifs of Hep1 were found to be incapable of rescuing growth defects in cells lacking Hep1 [46].

Human Hep1 stimulated the ATPase activity of mtHsp70 but this was not observed in yeast [19, 47–48]. Mutations of the key residues R81, H107, and D111 in human Hep1 decreased the binding affinities for HSPA9 [23]. Furthermore H107 played a critical role in stimulating the ATPase activity of HSPA9 [23]. Interestingly two of these residues are conserved in PfHep1 (H255 and D259), while R81 is replaced with K229 (Fig 1A). In yeast Hep1/Zim17, residues R106, H107 and D111have been shown to play a critical role in interactions with mtHsp70 [21]. Nuclear magnetic resonance (NMR) structural elucidations have shown that Zim17 has an L-shape with the two zing-finger motifs located at the end of the L sandwiched by two anti-parallel beta-sheets [21]. The zf-DNL of yeast Hep1 was used as a template to model the zf-DNL of PfHep1 and the overall structure of PfHep1 resembled that of yeast Hep1 with the cysteine residues found at the ends of two anti-parallel beta-sheets (Fig 1B and 1C).

### Aggregation of PfHsp70-3 is prevented by PfHep1 co-expression

**Fig 2:**
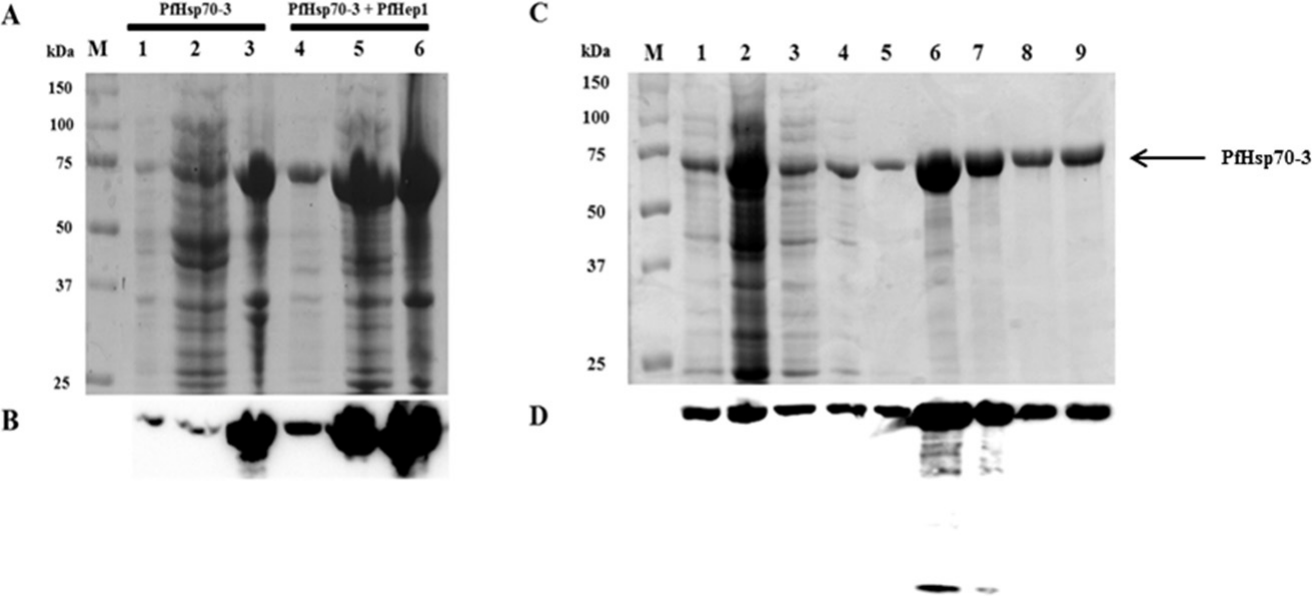
PfHep1 enhanced the solubility of PfHsp70-3 and facilitated native purification. (**A**) SDS-PAGE (10%) analysis of the solubility of PfHsp70-3 in the presence and absence of PfHep1. Lane M: protein markers in kDa, lane 1: *E*. *coli* BL21(DE3) [pQE30-PfHsp70-3] 4 hrs post IPTG induction (total protein), lanes 2–3: supernatant and pellet fractions of cells harvested and lysed 4 hrs post IPTG induction, lane 4: *E*. *coli* BL21(DE3) [pQE30-PfHsp70-3; pACYCDuet1-PfHep1] 4 hrs post IPTG induction (total protein), lanes 5–6: supernatant and pellet fractions from lysed cells co-transformed with pQE30-PfHsp70-3 and pACYCDuet-1-PfHep1 4 hrs post IPTG induction. (**B**) Western analysis using anti-His antibody. (**C**) SDS-PAGE (10%) analysis of the purification of PfHsp70-3, after co-expression with PfHep1, by nickel affinity chromatography. Lane M: protein markers in kDa, lane 1: *E*. *coli* BL21 (DE3) [pQE30-PfHsp70-3; pACYCDuet1-PfHep1] 4hrs post IPTG induction, lane 2: fraction unbound to the cOmplete His-tag purification resin, lanes 3–5: washes containing 50 mM imidazole, lanes 6–8: elutions of PfHsp70-3 and PfHep1 using 750 mM imidazole, lane 9: bead fraction. (**D**) Western analysis for detection of PfHsp70-3 (top) and PfHep1 (bottom) using anti-His antibodies.

The coding sequences of PfHsp70-3 and PfHep1 were inserted into pQE30 expression vectors without the mitochondrial signal sequences. PfHep1 was also inserted into pACYCDuet1 for the purposes of co-expression with PfHsp70-3. Induction of PfHsp70-3 with PfHep1 in *E*. *coli* cells was monitored over 5 hours and after overnight growth; western analysis revealed that both proteins were produced with lower levels of PfHep1 (data not shown). Examining the effect of PfHep1 co-expression with PfHsp70-3 revealed that it substantially enhanced the solubility of PfHsp70-3 (Fig 2A and 2B, lane 5). PfHsp70-3 alone was virtually insoluble, as seen by the prominence of the protein in the insoluble pellet (Fig 2A, lane 3) compared to the soluble fraction (lane 2). The solubility of PfHsp70-3 facilitated its native purification using nickel affinity chromatography without the need for denaturants such as urea. Some PfHsp70-3 was removed during the wash steps (Fig 2C, lanes 3–5) and a low concentration of PfHep1 (35 kDa) co-eluted with PfHsp70-3 (70.4 kDa) in elutions 1 and 2, however PfHep1 was not detected in elution 3 (Fig 2C and 2D, lanes 6–8).

**Fig 3:**
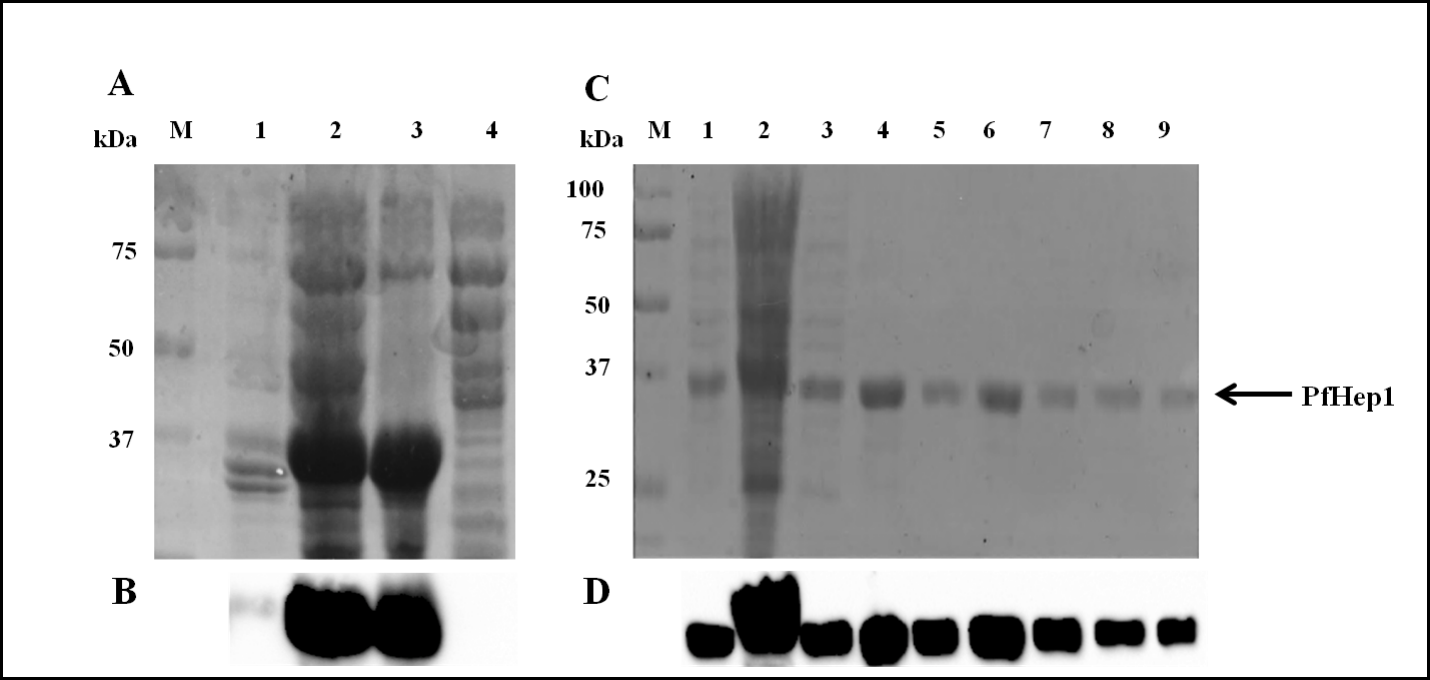
Solubilization and purification of PfHep1. (**A**) SDS-PAGE (10%) analysis of the solubility of PfHep1 before and after addition of sarcosyl. Lane M: protein markers in kDa, lanes 1–2: supernatant and pellet fractions of cells not treated with sarcosyl, lanes 3–4: supernatant and pellet fraction of cells treated with 3% sarcosyl. (**B**) Western analysis using anti-His antibody. (**C**) Purification of PfHep1 after solubilization with 3% sarcosyl, lane M: protein markers in kDa, lane 1: *E*. *coli* M15 ([pREP4]; pQE30-PfHep1) 4 hrs post IPTG induction, lane 2: fraction unbound to the cOmplete His-tag purification resin, lanes 3–5: washes using 50 mM imidazole, lanes 6–8: elutions of PfHep1 using 750 mM imidazole, and lane 9: bead fraction. (**D**) Western analysis for detection of PfHep1 using anti-His antibodies.

Human Hep1 displayed the features of a Type I J-protein and suppressed the aggregation of rhodanese [32]. To determine if PfHep1 performed a similar aggregation suppression role, the recombinant protein was purified after heterologous expression in *E*. *coli* [pQE30-PfHep1] cells. PfHep1 was initially found to be insoluble (Fig 3A and 3B, lanes 2 and 3), such that purification required the addition of the ionic detergent sarcosyl to the lysis buffer to solubilise the protein, prior to purification by nickel-affinity chromatography. Some PfHep1 was removed during the wash steps (Fig 3C, lanes 3–5) and PfHep1 was successfully eluted (Fig 3C, lanes 6–8). To our knowledge, the orthologues of PfHep1 are soluble and can be purified under native conditions. The insolubility of PfHep1 is probably due to the challenges associated with the heterologous expression of soluble and functional plasmodial proteins. Codon bias due to the AT-rich genome of *P*. *falciparum* can lead to poor protein expression levels in *E*. *coli* [49]. However, codon optimisation does not ensure expression as a large scale screen involving the heterologous expression of numerous *P*. *falciparum* genes revealed that a quarter of the codon optimised proteins remained insoluble [50]. The reason for this failure to express in a soluble form is unclear but could be attributable to the presence of long repeats of asparagine residues, resulting in a propensity of these proteins to form insoluble aggregates when heterologously expressed [44, 50].

### PfHep1 prevents thermally induced aggregation of PfHsp70-3

**Fig 4:**
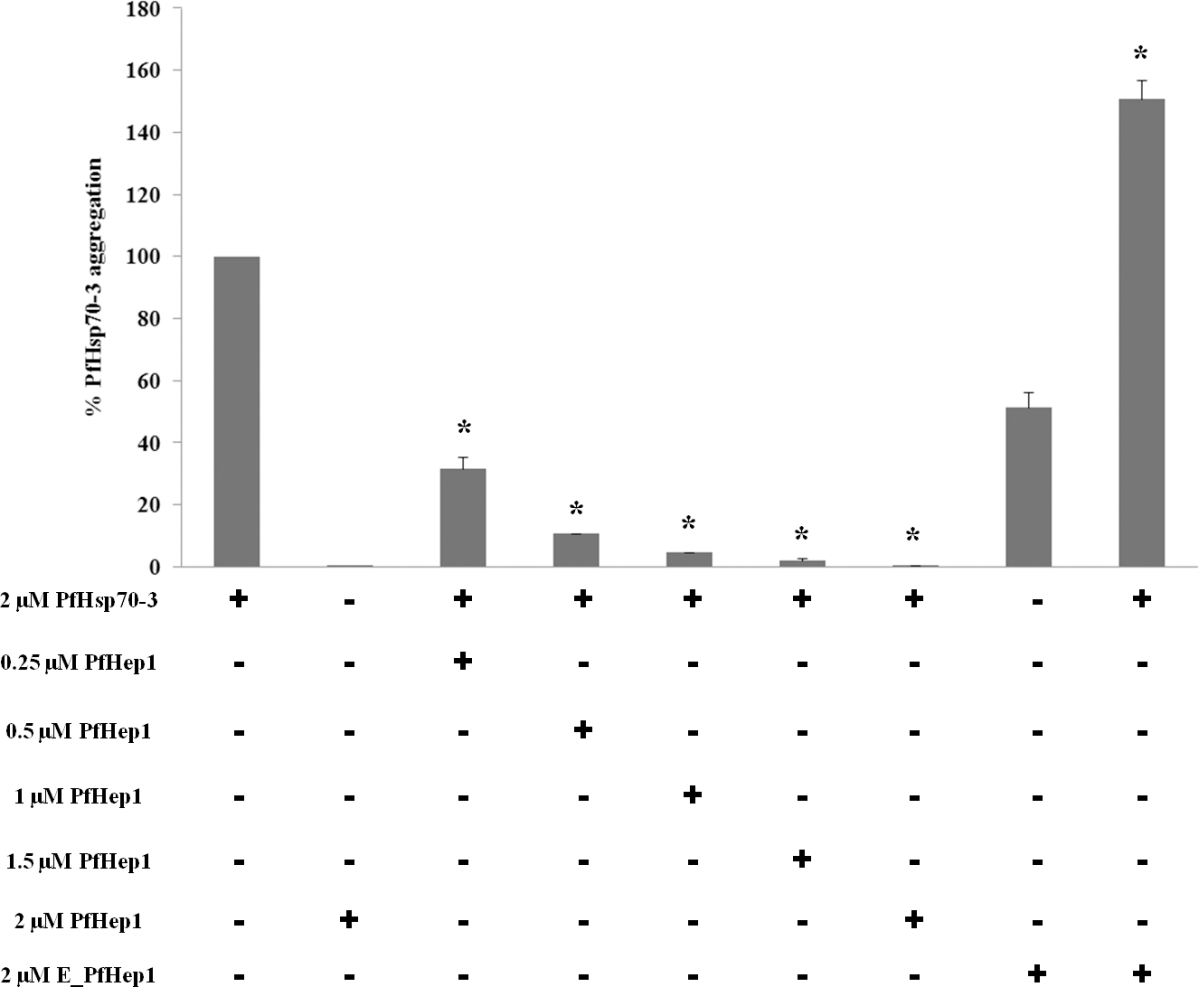
PfHep1 prevented the thermal aggregation of PfHsp70-3. The thermal aggregation of PfHsp70-3 (2 μM) was initiated by incubation at 50°C for 30 min and monitored by light scattering at 360 nm. Varying concentrations of PfHep1 were added to PfHsp70-3. PfHep1 suppressed the aggregation of PfHsp70-3 in a dose-dependent manner, with equimolar concentrations of PfHep1 and PfHsp70-3 resulting in complete aggregation suppression. PfHep1 after chelation of zinc ions by EDTA (E_PfHep1) aggregated and consequently was unable to suppress the aggregation of PfHsp70-3. The combined data of three independent experiments conducted in triplicate on three independent batches of protein are shown. The bars represent standard deviations. A statistically significant difference between a reaction and PfHsp70-3 alone is indicated by * (p>0.05) above the reaction using a Student’s t-test.

PfHsp70-3 aggregated at 50°C which enabled us to assess the ability of PfHep1 to suppress its thermally induced aggregation. PfHep1 suppressed the aggregation of PfHsp70-3 in a dose-dependent manner, with equimolar concentrations displaying complete suppression of aggregation of PfHsp70-3 (Fig 4). Similar results were obtained for *Leishmania braziliensis* Hep 1 (LbHep1), which suppressed the thermal aggregation of *L*. *braziliensis* mtHsp70 (LbmtHsp70) [39]. PfHep1 alone did not aggregate, while EDTA-treated PfHep1 (E-PfHep1; zinc ions removed) did aggregate under the assay conditions (Fig 4). E-PfHep1 failed to prevent the thermal aggregation of PfHsp70-3, and an additive effect was observed as the percentage aggregation was due to aggregated PfHsp70-3 and aggregated E-PfHep1 (Fig 4). EDTA has been shown to destabilize the structural integrity of LbHep1 probably by chelating zinc ions that in turn weakens the secondary and tertiary structure of the protein [50]. E-PfHep1 aggregated at 50°C and this was probably due to disruption of the structure after the removal of zinc ion. Zinc ions were found to be essential for maintaining the overall secondary structure of yeast Hep1 and LbHep1 [22,39].

### PfHep1 did not prevent thermally induced aggregation of malate dehydrogenate or citrate synthase

**Fig 5:**
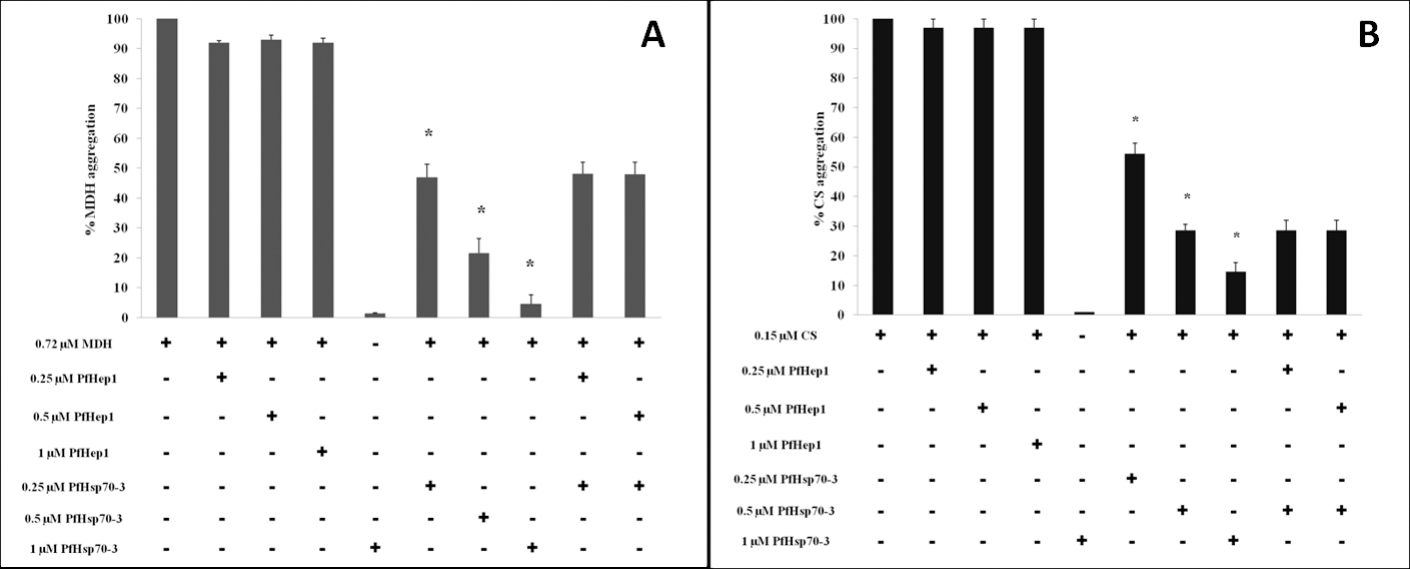
PfHep1 did not prevent the aggregation of MDH or citrate synthase. (**A**) MDH aggregation suppression assays were initiated by the addition of 0.72 μM MDH to assay buffer at 45°C for 30 min and monitored by light scattering at 360 nm. Varying concentrations of PfHep1 resulted in less than 10% suppression of MDH aggregation. (**B**) 0.15μM of citrate synthase was incubated at 45°C for 30 mins in both the absence and presence of varying concentrations of PfHep1 and PfHsp70-3. PfHep1 resulted in less than 3% suppression of CS aggregation. PfHsp70-3 suppressed the aggregation of both MDH and CS in a dose-dependent manner. PfHep1 did not enhance the ability of PfHsp70-3 to suppress MDH or CS aggregation and did not display intrinsic co-chaperone activity. The combined data of three independent experiments conducted in triplicate on three independent batches of protein are shown for both MDH and CS. The bars represent standard deviations. A statistically significant difference between a reaction and MDH alone is indicated by * (p>0.05) above the reaction using a Student’s t-test. The addition of PfHep1 to PfHsp70-3 and substrate did not produce a statistically different decrease in aggregation compared to PfHsp70-3 and substrate.

To determine if the prevention of aggregation by PfHep1 is specific to PfHsp70-3, it was necessary to assess its ability to suppress the aggregation of proteins that are known to aggregate thermally, such as MDH and citrate synthase. The addition of varying concentrations of PfHep1 resulted in less than 10% aggregation suppression of MDH and citrate synthase, while PfHsp70-3 suppressed the aggregation of MDH and citrate synthase in a dose-dependent manner (Fig 5A and 5B). PfHsp70-3 did not aggregate under the assay conditions (Fig 5), neither did PfHep1 (data not shown). PfHep1 did not enhance the ability of PfHsp70-3 to suppress aggregation of either MDH or citrate synthase (Fig 5A and 5B). Similarly, LbHep1 could not prevent the aggregation of two model proteins, MDH and Luc, but was specific for LbmtHsp70 [39].

## Conclusion

We have shown for the first time that PfHep1 is required for maintaining the solubility and thereby the activity of PfHsp70-3. Our study has indicated that PfHep1 functions as a specialised co-factor that facilitates the folding of PfHsp70-3. PfHep1 is larger than its orthologues and there is no sequence conservation outside of the zf-DNL. The zinc binding domain of PfHep1 was predicted to contain many of the conserved amino acid residues important for its interaction with PfHsp70-3. Not surprisingly, PfHsp70-3 was insoluble when heterologously expressed in *E*. *coli* cells, as many eukaryotic mtHsp70s are insoluble. We did not anticipate that PfHep1 would be insoluble and this may be due to plasmodial proteins being notoriously difficult to express heterologously in a soluble form, even after codon-optimization [50]. The co-expression of PfHep1 enhanced the expression and solubility of PfHsp70-3. The co-expression of PfHep1 and PfHsp70-3 facilitated the production of soluble and functional PfHsp70-3 that enables further biochemical characterisation of this chaperone in future studies. At high temperatures, beyond that of the human host, PfHep1 suppressed the aggregation of PfHsp70-3 but not other aggregation-prone proteins, such as MDH or citrate synthase. Furthermore, PfHep1 did not enhance the aggregation suppression activities of PfHsp70-3. Taken together, these data suggest that PfHep1 is not a co-chaperone of PfHsp70-3, but rather a specific co-factor to prevent its self-aggregation.

To our knowledge, human Hep1 is the only Hep protein that has been demonstrated to display the features of a J-protein as it stimulated the ATPase activity of mtHsp70 and functioned as a holdase by binding unfolded proteins such as rhodanese [47–48]. The sequence alignment revealed that the region downstream of the zf-DNL or C-terminal sub-domain, is longer in both humans and yeast with a lack of sequence identity between the two species (Fig 1A). There is evidence to suggest that the C-terminal sub-domain of human Hep1 is responsible for regulating the activity of zf-DNL and conferring co-chaperone activity [51]. An examination of the direct functional effects of Zim17 on mtHsp70 in the cell indicated a novel role of Zim17 in assisting its interaction with client proteins in a J co-chaperone-dependent manner [52]. There is also the proposition that Zim17 functions as a “fractured” J-protein that provides a zinc finger domain to Type III J-proteins for substrate binding [53]. However, this remains to be experimentally elucidated.

The mechanism of action of human and yeast Hep1 appears to be different from that of other orthologues, and further studies are required to understand this differentiation. A greater understanding of Hep proteins from different organisms is required to determine whether or not they have the properties of *bona fide* co-chaperones. Thus future mechanistic studies on PfHep1-PfHsp70-3 could include determination of the ability of PfHep1 to stimulate the ATPase activity of PfHsp70-3 and identification of potential J protein co-chaperones in the mitochondrial matrix. In view of the fact that LbmtHsp70 plays a key role in the adaptation of the parasite in the host, disrupting the interaction between LbHep1 and LbmtHsp70 is a potential target for new therapies [39]. Likewise, abrogating the specific partnership between PfHep1 and PfHsp70-3 may also be a target for new antimalarials.
